# Supplementary figures and images for: Lessons from the meiotic recombination landscape of the ZMM deficient budding yeast Lachancea waltii
Source: PLoS Genet. 2023 Jan 6;19(1):e1010592. doi: 10.1371/journal.pgen.1010592 (PMC9851511; doi:10.1371/journal.pgen.1010592)

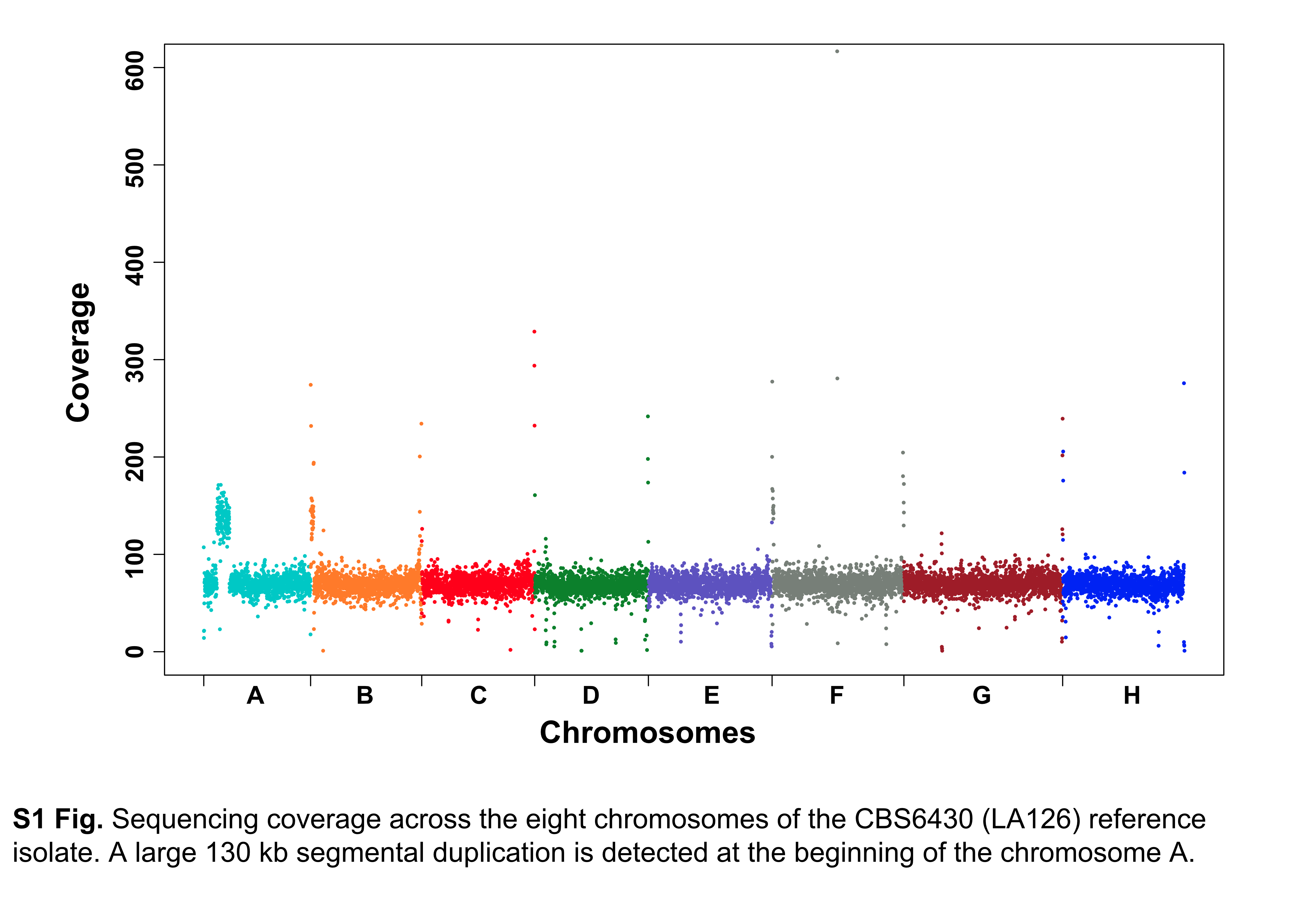

Supplement: S1 Fig — A large 130 kb segmental duplication is detected at the beginning of the chromosome A. (TIF) [file pgen.1010592.s012.tif]

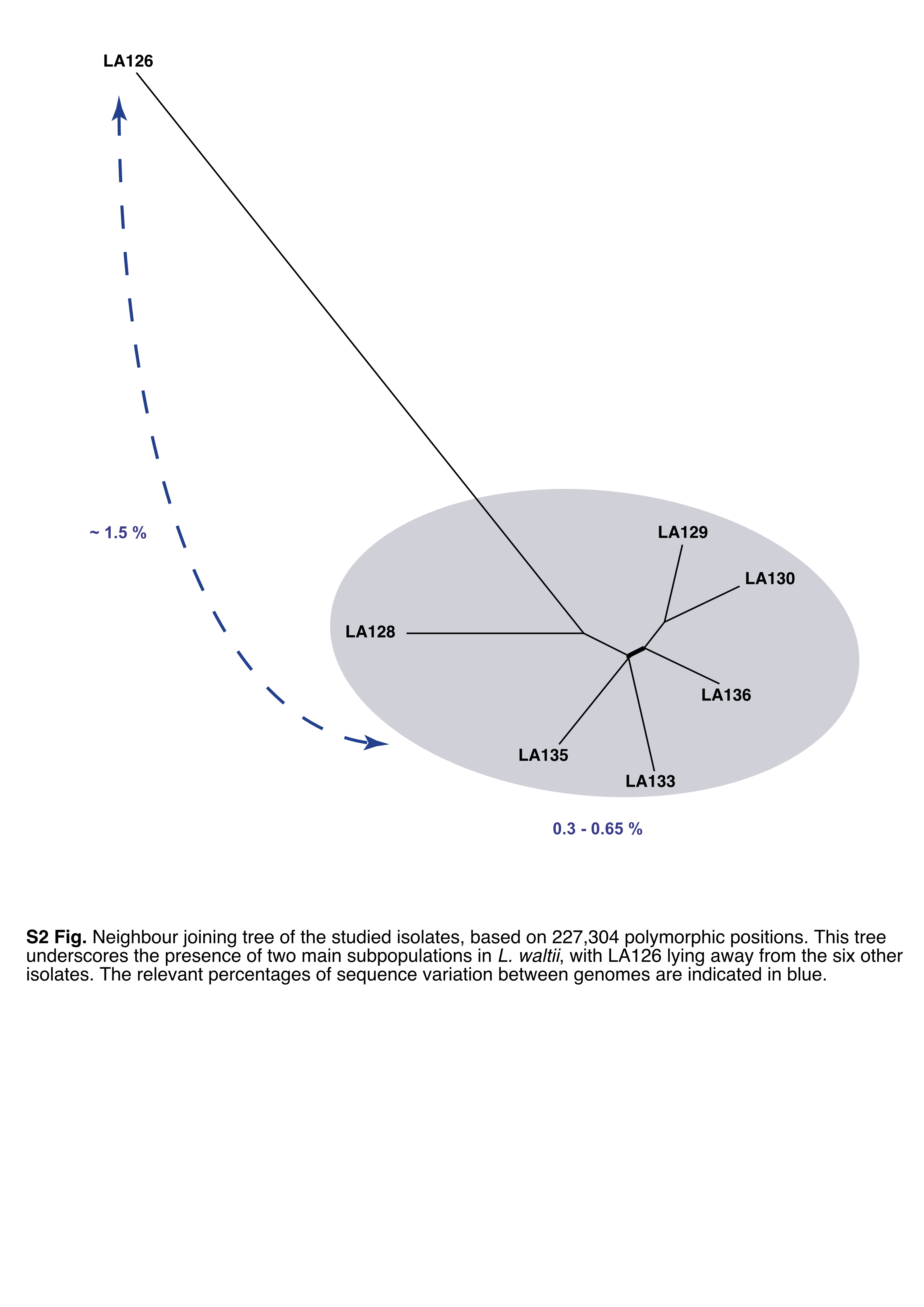

Supplement: S2 Fig — This tree underscores the presence of two main subpopulations in L. waltii, with LA126 lying away from the six other isolates. (TIF) [file pgen.1010592.s013.tif]

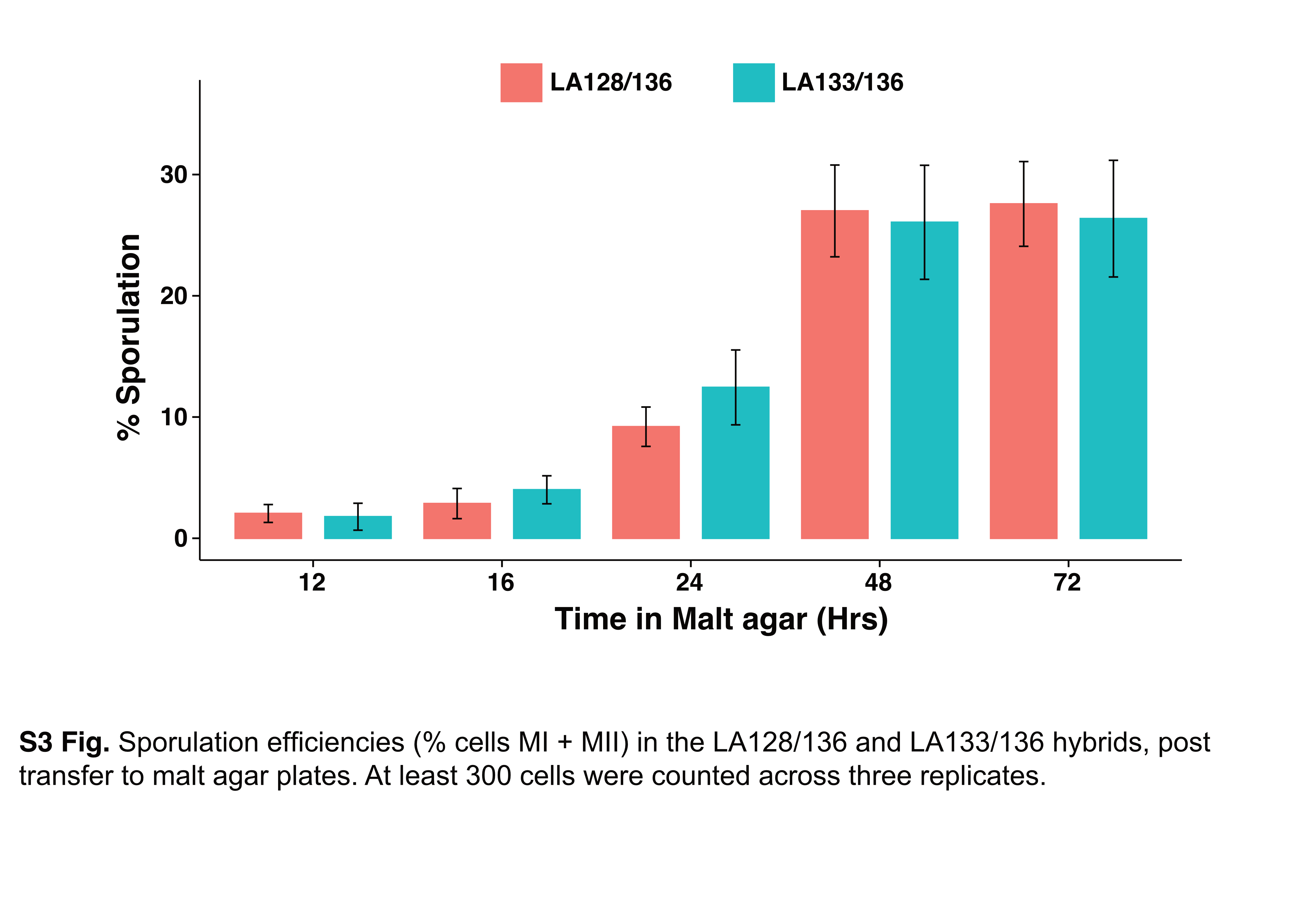

Supplement: S3 Fig — At least 300 cells were counted across three replicates. (TIF) [file pgen.1010592.s014.tif]

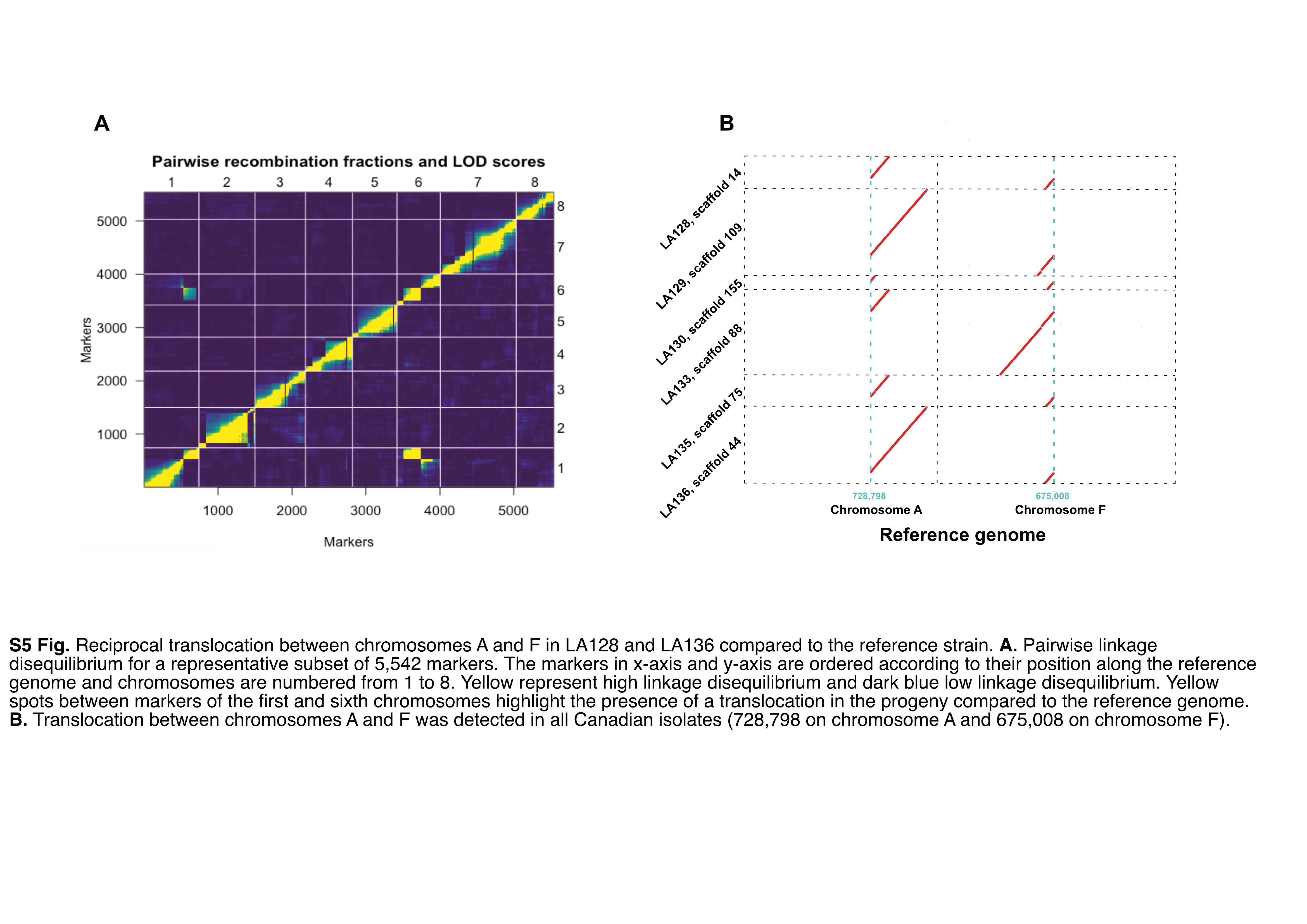

Supplement: S5 Fig — A. Pairwise linkage disequilibrium for a representative subset of 5,542 markers. The markers in x-axis and y-axis are ordered according to their position along the reference genome and chromosomes are numbered from 1 to 8. Yellow represents high linkage disequilibrium and dark blue low linkage disequilibrium. Yellow spots between markers of the first and sixth chromosome highlight the presence of a translocation in the progeny compared to the reference genome. B. Translocation between chromosomes A and F was detected in all Canadian isolates (728, 798 on Chromosome A and 675, 008 on Chromosome F). (TIF) [file pgen.1010592.s016.tif]

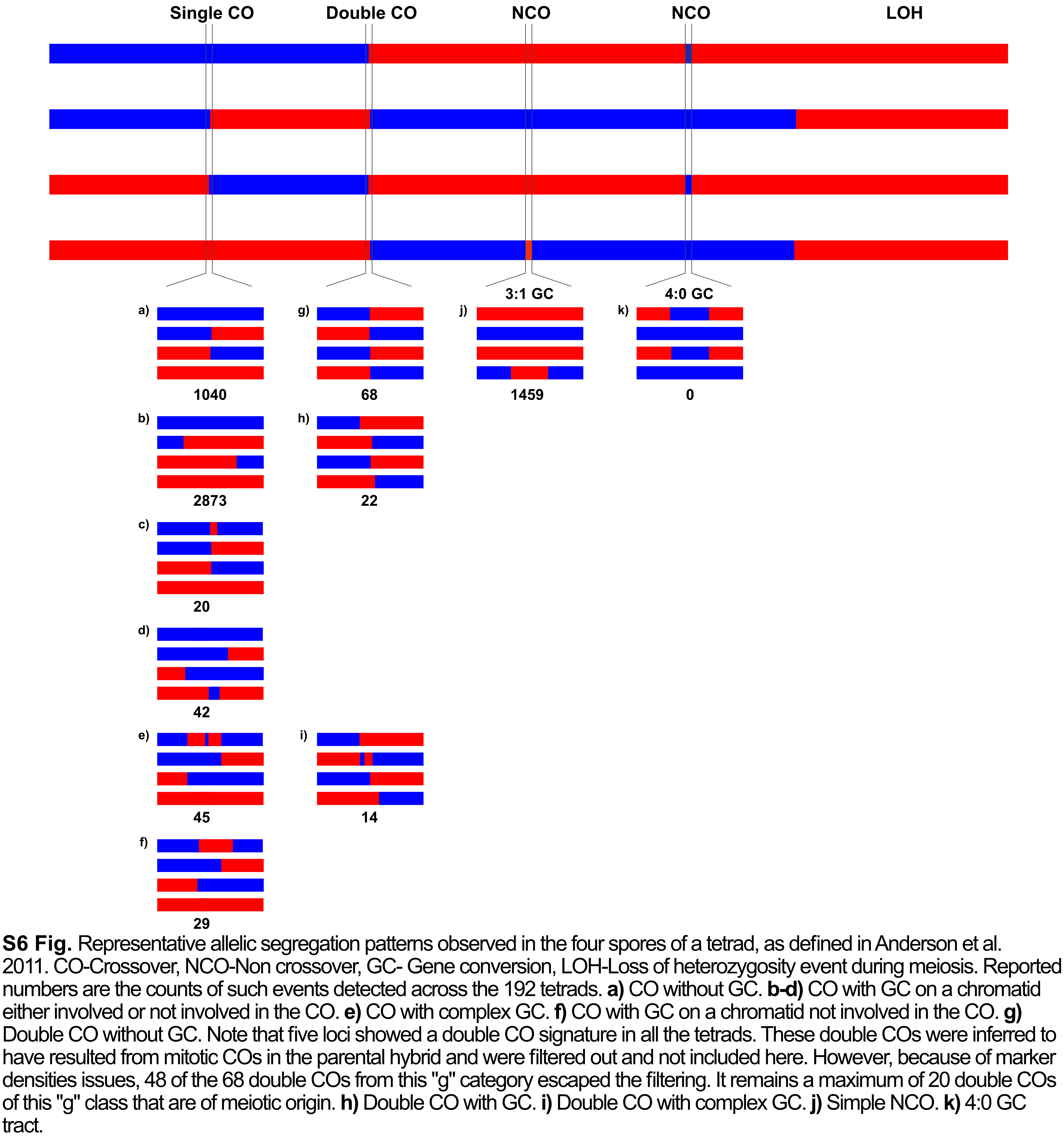

Supplement: S6 Fig — CO-Crossover, NCO-Non crossover, GC- Gene conversion, LOH-Loss of heterozygosity event during meiosis. Reported numbers are the counts of such events detected across the 192 tetrads. a) CO without GC. b-d) CO with GC on a chromatid either involved or not involved in the CO. e) CO with complex GC. f) CO with GC on a chromatid not involved in the CO. g) Double CO without GC. Note that five loci showed a double CO signature in all the tetrads. These double COs were inferred to have resulted from mitotic COs in the parental hybrid and were filtered out and not included here. However, because of marker densities issues, 48 of the 68 double COs from this "g" category escaped the filtering. It remains a maximum of 20 double COs of this "g" class that are of meiotic origin. h) Double CO with GC. i) Double CO with complex GC. j) Simple NCO. k) 4:0 GC tract. (TIF) [file pgen.1010592.s017.tif]

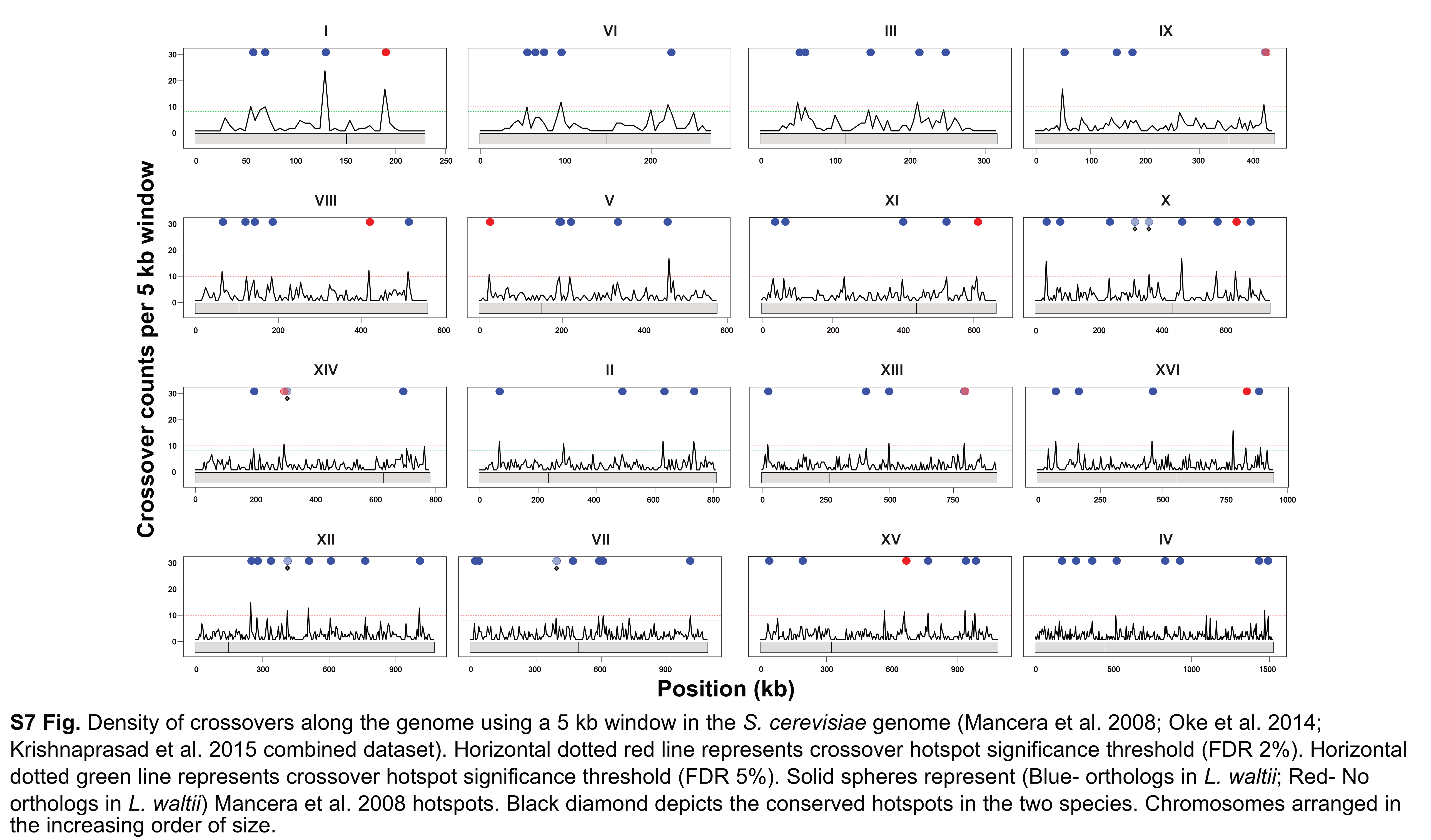

Supplement: S7 Fig — Horizontal dotted red line represents crossover hotspot significance threshold (FDR 2%). Horizontal dotted green line represents crossover hotspot significance threshold (FDR 5%). Solid spheres represent (Green- orthologs in L. waltii; Red- No orthologs in L. waltii) Mancera et al. 2008 hotspots. Black diamond depicts the conserved hotspot in the two species. (TIF) [file pgen.1010592.s018.tif]

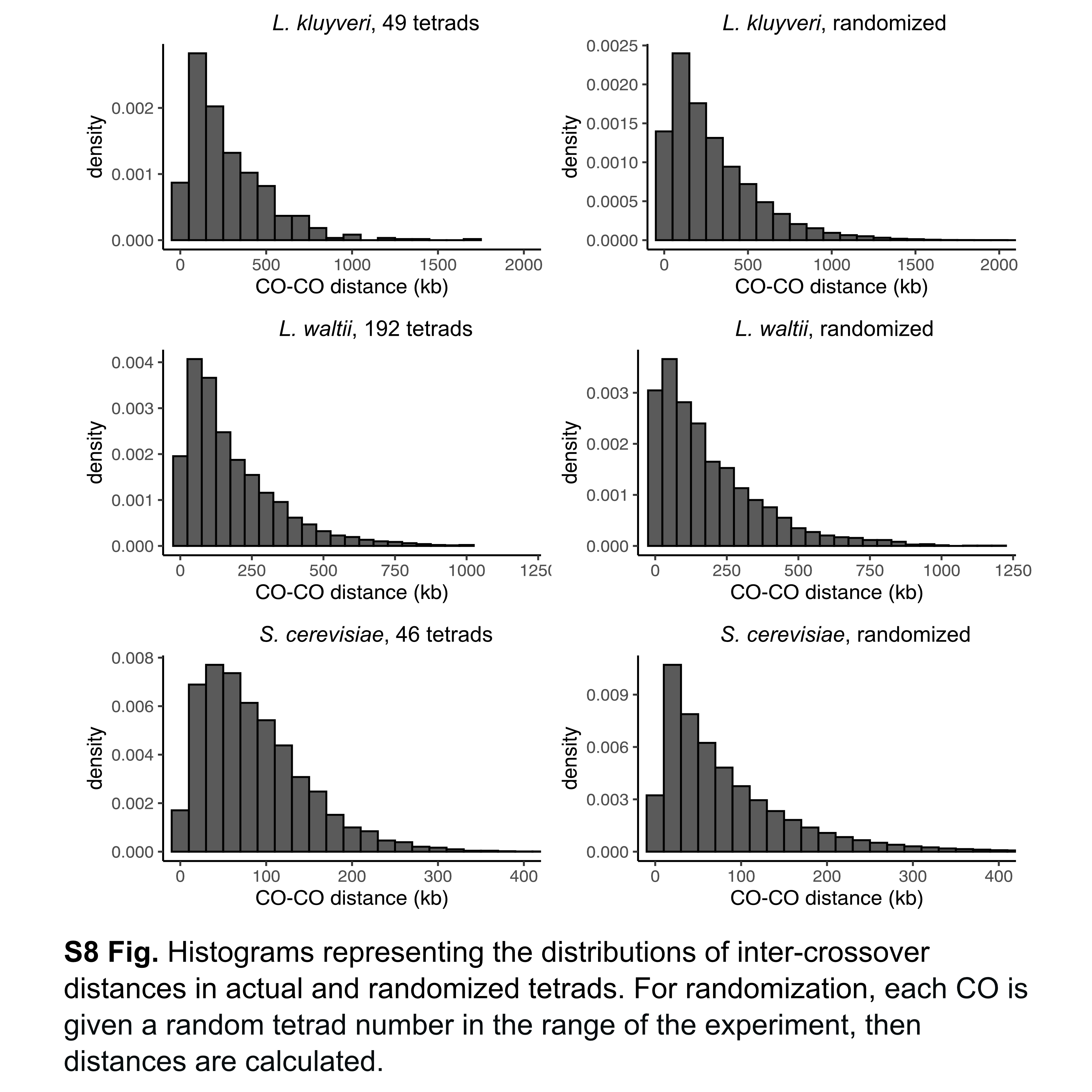

Supplement: S8 Fig — For randomization, each CO is given a random tetrad number in the range of the experiment, then distances are calculated. (TIF) [file pgen.1010592.s019.tif]
