# Supplementary material for: Lessons from the meiotic recombination landscape of the ZMM deficient budding yeast Lachancea waltii
Source: PLoS Genet. 2023 Jan 6;19(1):e1010592. doi: 10.1371/journal.pgen.1010592 (PMC9851511; doi:10.1371/journal.pgen.1010592)
Supplement: S9 Fig — (TIF) [file pgen.1010592.s020.tif]

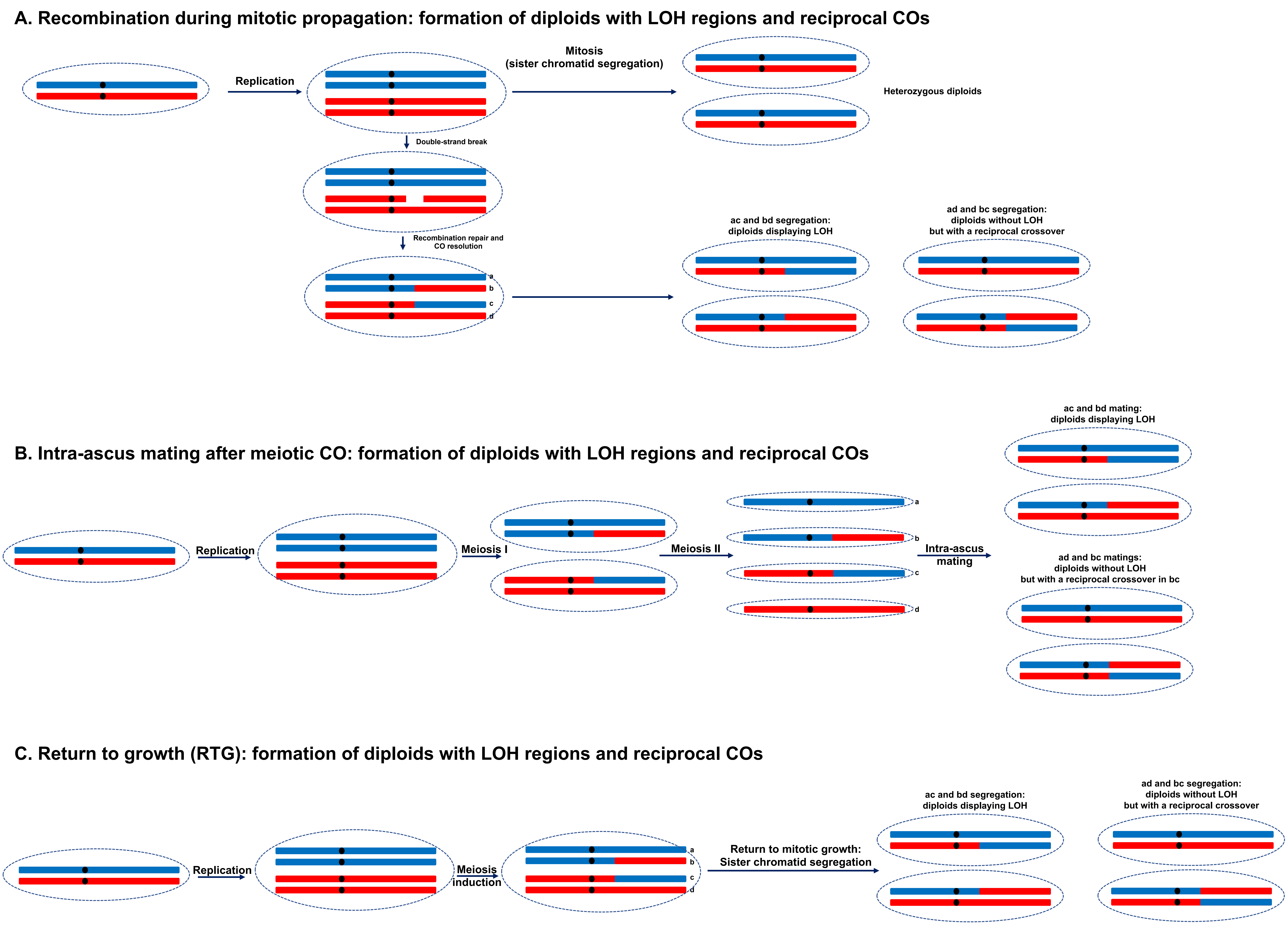

A. Recombination during mitotic propagation: formation of diploids with LOH regions and reciprocal COs
Replication
Mitosis
(sister chromatid segregation)
Heterozygous diploids
Double-strand break
Recombination repair and
CO resolution
a
b
c
d
ac and bd segregation:
diploids displaying LOH
ad and bc segregation:
diploids without LOH
but with a reciprocal crossover
B. Intra-ascus mating after meiotic CO: formation of diploids with LOH regions and reciprocal COs
Replication
Meiosis I
Meiosis II
a
b
c
d
Intra-ascus
mating
ac and bd mating:
diploids displaying LOH
ad and bc matings:
diploids without LOH
but with a reciprocal crossover in bc
C. Return to growth (RTG): formation of diploids with LOH regions and reciprocal COs
Replication
Meiosis
induction
a
b
c
d
Return to mitotic growth:
Sister chromatid segregation
ac and bd segregation:
diploids displaying LOH
ad and bc segregation:
diploids without LOH
but with a reciprocal crossover

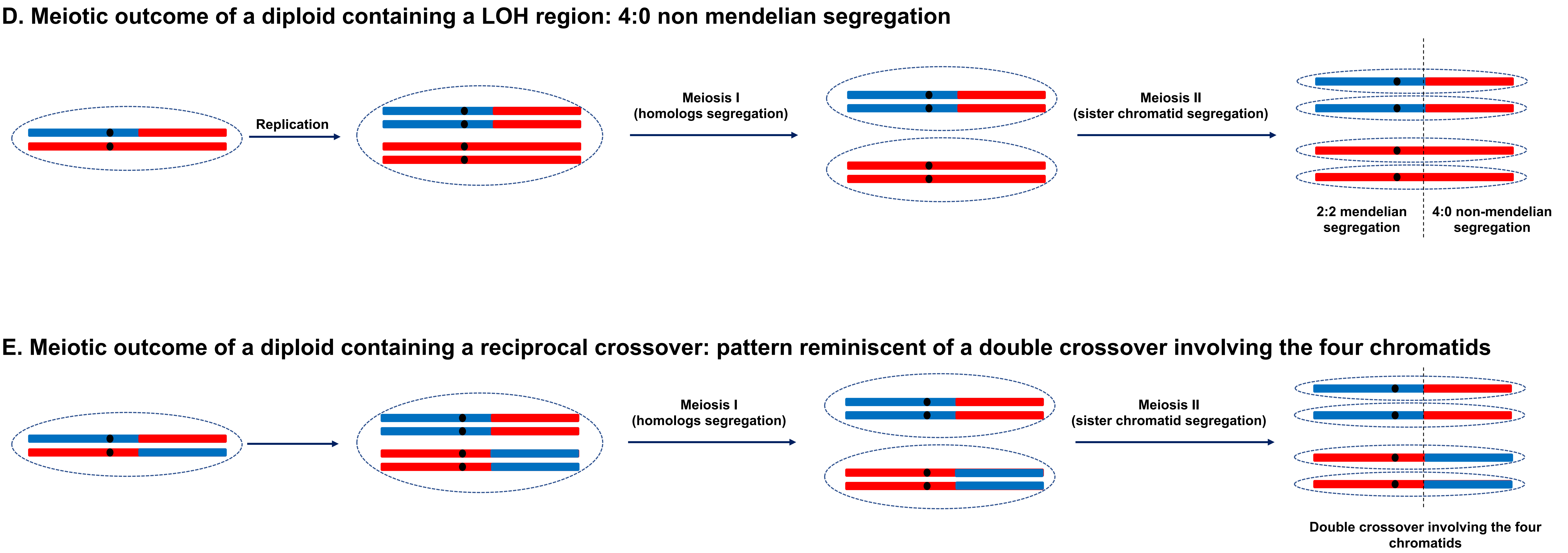

S9 Fig. Possible scenarios leading to LOH and reciprocal COs in the *L. waltii* hybrids and outcomes in *L. waltii* meiotic segregants

Recombination during mitotic propagation (A), intra-ascus mating (B) and return to growth (C) are three possible routes to generate LOH and concomitant reciprocal COs in *L. waltii* hybrids. LOH in *L. waltii* hybrids result in 4:0 meiotic segregation profiles (D). Reciprocal COs in *L. waltii* hybrids result in apparent meiotic double crossovers involving the four chromatids (E). For the sake of simplicity, only one recombination event is shown here. However, when multiple events occur in the same cell, a combination of LOH regions and reciprocal COs is generated, in agreement with the expected genotype from our starting *L. waltii* hybrid.
