# Supplementary material for: Lessons from the meiotic recombination landscape of the ZMM deficient budding yeast Lachancea waltii
Source: PLoS Genet. 2023 Jan 6;19(1):e1010592. doi: 10.1371/journal.pgen.1010592 (PMC9851511; doi:10.1371/journal.pgen.1010592)
Supplement: Attachment — Submitted filename: PLoS_Gen_Responses.docx [file pgen.1010592.s021.docx]

Dear Michael,

Thank you very much for the reviews on our manuscript titled “Lessons from the meiotic recombination landscape of the ZMM deficient budding yeast *Lachancea waltii*”. We appreciated the overall enthusiasm of the reviews as well as the constructive feedback. We have made changes to address all the points raised by reviewers and have revised the manuscript accordingly. Detailed changes and discussion are also provided in the responses below. We believe these changes have substantially improved the manuscript.

Best regards,

Bertrand and Joseph

**Editors comments**

You will note that reviewer 2 has suggested analysis of several mutants, in particular *mer3* and possibly *mlh3* and *mus81*, at least to the level of spore viability/sporulation efficiency. I agree that these studies, in particular of *Mer3*, would be important and would considerably add to the interest of the manuscript. However, I agree with reviewer 1 that the manuscript is of sufficient interest to not require this additional work, but again I think that the conservation of *Mer3* is very interesting and a preliminary characterization of *mer3∆* mutants, at least at the level of spore viability, would be quite interesting.

[R] Given our findings, we definitely plan to analyze several mutants, including *mer3*, *mlh3* and *mus81* to further dissect CO formation in *L. waltii*. However, we believe this is beyond the scope of the current work and we are considering doing so as part of a follow-up study.

My other comments are as follows:

1. Fig 1, what are the CO frequency units?

[R] The crossover frequency is the number of COs per 5 kb window. It is now indicated directly on the Y axis of the figure, and defined in materials and methods.

2. Page 5, 13 Since *Mlh2* appears to be absent but conversion tracts are cerevisiae-like, is there a functional *Pif1* homolog in waltii, since *Pif1* is required for the longer GC tracts seen in cerevisiae *mlh2* mutants.

[R] Very limited functional studies have been performed in *L. waltii*. Most gene functions are deduced from their homology to the reference *S. cerevisiae.* This is obviously only a proxy and would deserve experimental confirmation on a case-by-case basis, as for the *PIF1* gene. From the genome sequence analysis, the only thing we can say is that there are two *PIF1* homologs in *L. waltii*. Based on sequence similarity, LAWA0D02212g is likely the *S. cerevisiae* *YML061c* *PIF1* ortholog and LAWA0G13124g is likely the *S. cerevisiae* *YHR031c* *RRM3* ortholog. In conclusion, the most likely scenario is that a functional homolog of *PIF1* exists in *L. waltii*. In case it is recruited to meiotic DSBs as in *S. cerevisiae*, the Mlh1-2 function could be taken over by alternative players. In that respect, it is important to note that Mer3 itself can counteract Pif1 mediated extension, although to a lesser extent compared to the Mer3-Mlh1/2 complex (Vernekar et al. 2021).

3. Page 5. Suggest “Finally, consistent with the loss of…”

[R] This has been corrected as suggested.

4. Page 8. The Wilcoxon test is a paired-values test and is not appropriate here, as there are unequal numbers of GC_co_, and NCOs and they are not paired with each other. A Mann-Whitney test, which is appropriate, reports that the GCco and NCO distributions ARE significantly different (p<0.0001). Please note that both Wilcoxon and Mann-Whitney are tests of the entire distribution, not the median. It is possible for medians to be the same while distributions are different.

[R] Thank you for pointing the error out. We have corrected the text and stated p-values based on the Mann-Whitney U test< p=6.388e-11 >.

5. Page 8. Based on the frequency of E0s, if extrapolated to all chromosomes, what is the expected spore viability assuming no distributive distribution?

[R] Naive calculation using chromosome A for which we have a full recombination map, not altered by any LOH tract in the hybrid:

i. Chromosome A shows ca. 20% E0

ii. Assuming a similar E0 frequency for all chromosomes, which vary in size by less than two folds unlike in *S. cerevisiae* where chromosome sizes show much bigger variations, the expected frequency of an achiasmate chromosome per *L. waltii* meiosis is:

\# chromosome x E0 frequency per chromosome, that is 8 x 20% = 160%

iii. Since achiasmate chromosomes have 50% chance of disjoining improperly, the expected frequency that one chromosome segregates improperly per *L. waltii* meiosis is 160% x ½ = 80%

iv. In conclusion, the expected frequency that one chromosome segregates improperly per *L. waltii* meiosis largely exceeds the 30% of spore inviability, which supports the existence of an alternative mechanism to promote meiotic chromosome segregation.

6. Page 10. Suggest “L. waltii crossovers exhibit limited interference” or "*L. waltii* crossovers exhibit interference over only short distances"

[R] This has been changed as suggested to… “*L. waltii* crossovers exhibit interference over only short distances (up to 25 kb).”

8. Page 11. “a gamma law of shape = 1”. Do you mean that k = 1? Might be worth briefly explaining what k and theta are in the legend to Figure 3.

[R] in fact “gamma law of shape =1”, represents, k/γ=1. We have additionally elaborated on γ/⍬ in the figure 2 legends section.

**Reviewer #1**

The manuscript describes the genome-wide analysis of meiotic recombination via the sequencing of haplotypes arising in meiotic spores of an intra-specific hybrid of the relatively unexplored yeast species, *Lachancea waltii*. The dataset is extensive (192 meioses), although detailed analysis of the results is relatively limited—in part because there are limited other data with which to compare results with. Crossover (CO) and noncrossover (NCO) frequency are calculated, CO distributions are assessed, and the evolutionary conservation of recombination hotspot positions is discussed. The results are compared to observations made in two other yeast species (*S. cerevisiae*, and *L. kluyveri*), the former of which many meiotic experiments have been performed, and much mechanistic detail has been inferred.
Overall, the study is somewhat limited in the impact that this dataset can make, but the study represents a commendable foray into the exploration of meiotic recombination outside of the classic species *S. cerevisiae* and *S. pombe*. I have only a few comments that might help the authors to improve the understanding and impact of the study.

**Major comments**

1. General. I found the overall description of the LOH section (and the 4-strand double CO part), and in particular the parts referring to fig S3 (and Fig S3 itself), unclear. Perhaps break up into shorter paragraphs and add some helpful cartoon figures to explain the concepts/interpretations?

[R] We believe this refers to the discussion section since only this part refers to the 4-strand double COs. Supplementary figure S3 (now S4) depicts LOH regions (red shaded regions) observed on several diploids that we generated. We have now included supplementary figure S9, to describe possible scenarios leading to LOH outcomes in *L. waltii* hybrids. We also clarified the corresponding discussion. Importantly, unlike our initial claim, re-analyzing our recombination data revealed the existence of 4-strand double COs as expected from any known pathway leading to LOH in the hybrid.

2. For the parts about hotspot conservation, it would help if the general concept here was more clearly introduced and explained. Is it expected that hotspots will be conserved at the location of orthologous genes? If so, by what mechanism is the gene (not the gene function) thought to be regulating this?

[R] The concept of evolutionary stable recombination hotspots in the absence of PRDM9 is mentioned in the second paragraph of the introduction notably referring to Lam and Keeney 2015. The hypothesis is that the gene promoter itself, by being a nucleosome free genomic region, is a DSB hotspot independently of the gene function. Hence, it is expected that DSB hotspots and the resulting recombination hotspots are conserved at orthologous locations, and this is what we tested.

Detection of orthologous syntenic regions between *S. cerevisiae* and *L. waltii* is described in the methods section. For each *S. cerevisiae* recombination hotspot, orthologs of the five closest linked genes were searched in the annotated genome of the *L. waltii* reference strain CBS 6430. Synteny was considered conserved and suitable for hotspot comparison when more than two orthologs associated to the same hotspots were detected in the same region (separated by less than 10 kb). If the distance between two recombination hotspots was less than 5 kb, they were deemed to be conserved.

3. Fig 2. A & B. Please add R values and P value statistics to the linear correlations.

[R] Figures have been updated with R values and P-values.

4. Fig 2C. It would be helpful to replot these distributions normalized as a fraction of events so that the CO and NCO length distributions can be more easily compared.

[R] We updated figure 2C and plotted ‘event densities’ instead of ‘frequencies’.

5. Fig 3A. Is there a reason that the CoC curve is inverted compared to how it is has become to be plotted in the field (*i.e.* N. Kleckner)? What does it mean that the *L. waltii* data show negative interference at all distances? Unless I missed it, this was not discussed, but seems of potential interest.

[R] Either the CoC value is plotted (i.e., N. Kleckner) or the interference value ie 1-CoC (J. Fung, see. Anderson et al. PLoS Genetics 2015, Figure 2C).

Crossover interference is low but positive in *L. waltii* at the 25 kb inter-interval distance. It becomes negative only at longer inter-interval distances. We agree with this reviewer that, formally, the negative interference values could be compatible with concerted crossover formation. However, these interference values are rather weak with some of them close to zero that’s why we preferred not to discuss them further.

6.The authors suggest that short range positive interfrence may be due to DSB interference. If so, it may be expected that NCOs also show the same trend. Is the dataset deep enough to assess CoC between NCOs? It seems that with 192 meiosis it might be possible NCO distributions in a little depth (and perhaps CO-NCO relationships?).

Figure 1: Interference calculated for a bin size of 25 kb and inter-interval distance of 25 kb, blue: Inter event; orange-NCO-NCO.

[R] We calculated interference between NCOs only, and between COs and NCOs combined. i. The interference values for NCOs only oscillate between zero and negative values suggesting no interference between NCOs in *L. waltii* similar to *S. cerevisiae* (Mancera et al. 2007).

ii. We do observe interference between events when COs and NCOs are combined. As for interference between COs only, interference is exhibited over 25 kb and rapidly decays thereafter, probably because COs outnumber NCOs. Interestingly, the interference value is higher when COs and NCOs are combined compared to COs only, notably at the 25 kb inter-interval distance. This tends to reinforce the notion of interference between DSBs.

7. Fig 3C. I’m not sure it makes sense to overlap these curves given that:
A) the CO frequency varies greatly between each strain, and thus the CO-CO distribution will vary even if interference does not.
B) If axis length is the relevant measure (of interference), without knowing the relative axis lengths between the different species, it is not clear how one would interpret the plot. Perhaps this point can be better highlighted/acknowledged?
C) Could each dataset be compared to a random (expected) distribution to determine by how much each CO-CO distribution deviates from random? Stating the gamma shape parameters in text and figure here would also help.

[R] We have now presented a comparison of each dataset to a random distribution (*i.e.* each CO is given a random tetrad number in the range of the experiment, then distances are calculated) (Figures 3C; S8).

8. Fig S6: Were there no events that had greater complexity that these examples? Is that surprising and/or consistent with data from *S. cerevisiae*?

[R] The first version of Fig S6 was a simplified view of the recombination events patterns. The revised version of this figure now includes a deeper level of resolution of the recombination events patterns according to the classification of Anderson et al 2011. This shows that *L. waltii* contains complex events such as those involving three chromatids. However, these complex events represent only ~1.5% in *L. waltii* while they represent ~10% of the events in *S. cerevisiae* (Mancera et al 2008, FigS3-4).

9. Fig S7: Minor: Suggest replotting with chromosomes ordered either by size or by chromosome number.

[R] We replotted as suggested with chromosomes arranged in ascending order of size.

10. Minor. The text refers to the concept that CO interference in *S. cerevisiae* “relies” on Top2 and Red1. This seems to overstate the observations of this prior study where mutations in those factors only reduced but did not abolish CO interference.

[R] This has been changed in the main text as suggested.

11. Minor: When describing loop sizes in *S. cerevisiae*, as well as the recent Hi-C estimates, it would make sense to also refer to much earlier EM estimates such as Moens & Pearlman 1998 etc.

[R] Yes indeed. We now include the reference.

**Reviewer #2**

In this article, the authors describe the meiotic recombination profile of a budding yeast species, *Lachancea waltii*, that diverged from *S. cerevisiae* before whole genome duplication, and lacks most of the ZMM genes involved in meiotic crossover formation, contrary to *S. cerevisiae* and another closely related species, *Lachancea kluyveri* that they studied previously.
For this, they established the genomic diversity of several waltii isolates, and chose to analyze two hybrid diploids that differ at many polymorphic sites, which allows monitoring meiotic recombination events. In addition to this genomic characterization, they establish the proper conditions to trigger sporulation, and notice that for unexplained reasons, the parental diploids already exhibit a high number of loss of heterozygosity (LOH) events, precluding the analysis of a part of the genome (about half).
Nevertheless, they managed to analyze the meiotic segregation profile of 192 tetrads, which they compare to that of budding yeast *S. cerevisiae*, and the “ZMM-proficient” *L. kluyveri* that the analyzed previously.
Their main conclusions are that like *L. kluyveri*, *L. waltii* exhibits less crossovers per genome than *S. cerevisiae*, that crossover density does not inversely correlate with chromosome size, contrary to *S. cerevisiae*, and that, unexpectedly, crossovers still show interference, despite the strain missing most ZMM genes. In addition, the crossovers hotspots that they determined thanks their high number of tetrads analyzed, do not match with those of *S. cerevisiae*, even in large syntenic blocs.

Globally, the paper is well written, the genomic analyses are well performed with sufficient statistical power due to the large number of tetrads analyzed, and the appropriate controls are provided. However, it is mainly descriptive, and does not provide any experiment to support the proposed explanations to the observed recombination properties.

[R] Thank you for appreciating our work and efforts. The power and relevance of the work is that it relies on “natural mutagenesis” to address mechanistic questions. While a similar study in *S. cerevisiae* would have implied the generation of multiple mutants, potentially associated with meiotic viability issues, here, Zip2, 3, 4, Msh4, Msh5, Spo16 and Mlh2 have been naturally lost after the speciation from *L. kluyveri*. In terms of mechanisms, our work reveals a very weak CO interference in the absence of the ZMM pathway. On one hand, as expected, this supports that the ZMM pathway is responsible for most of CO interference in *L. waltii*, a mechanism first described more than a century ago, but whose molecular basis remains elusive. On the other hand, this shows that in the absence of the ZMM pathway there is another source of weak CO interference, likely the patterning of CO precursors, which is generally overlooked. In addition, our work revealed that *L. waltii* gene conversion tract sizes are like those of *S. cerevisiae* despite the absence of Mlh2 known to restrict their extent. Finally, our work revealed an unanticipated mechanism responsible for the frequent formation of LOH within *L. waltii* hybrids which molecular bases still need to be determined. Hence, our work is *de facto* not “entirely descriptive”, although it contains additional “more descriptive” results. Such additional results are typical from the genetics field and include:

**(i)** The genetic diversity within the *L. waltii* species

**(ii)** The first *L. waltii* partial meiotic recombination map and

**(iii)** The lack of conservation of CO hotspots between *L. waltii,* *L. kluyveri* and *S. cerevisiae*.

**Major Comments**

1. Since MER3 and the SC gene ZIP1 are the only remaining ZMM genes in the waltii species, I think an essential experiment, which should be relatively straightforward, would be to perform a MER3 deletion, and at least analyze the spore viability and sporulation efficiency, to determine if, on its own, Mer3 is at all involved in meiotic recombination in this species. This is especially relevant since the authors mention that recombination tracts are of a similar size as those of *S. cerevisiae*, despite having lost the MLH2 gene. The Mlh1-Mlh2 heterodimer was described in budding yeast to limit the size of recombination events through it recruitment by Mer3. If meiotic recombination in waltii proceeds in a zmm-independent way, involving structure-specific nucleases such as Mus81-Mms4, Mlh2 would not be expected to influence the length of meiotic recombination events. It is therefore important to determine if Mer3 has any role in meiotic recombination on waltii. Also, a recent preprint on Biorxiv described that in addition to interacting with Mlh1-Mlh2 and limiting DNA synthesis, Mer3 may antagonize the action of the Sgs1 helicase to ensure interfering crossover formation (Altmannova et al.). It may therefore participate, even as the sole ZMM, to promote some interfering crossovers.

[R] Thank you for the comments and suggestions. We agree and believe that more exploration on *mer3*, *mlh3*, and *mus81* would provide more insights to understand how COs are formed in *L. waltii*. But we think it goes outside the purview of the current work and would be more suitable for a follow-up.

In addition, it is important to remind that in *S. cerevisiae*, Mer3/Mlh1-2 restrain the extent of both COs and NCOs-associated gene conversions (Duroc et al 2017). Given that most NCOs arise independently of the ZMM pathway, this shows that Mer3 exerts some of its functions outside the ZMM pathway and on most recombination intermediates. Therefore, based on this *S. cerevisiae* model, one would have expected that the loss of Mlh2 would influence gene conversion tract lengths even in *L. waltii* that lacks the ZMM pathway.

2. Page 6, 2nd paragraph: Figures S3 and S4 numbers should be inverted, since current S4 is cited before current S3.

[R] This has been corrected as suggested.

3. Page 8: for clarity, the authors should better explain that the infer a total of 34 Cos and 12.5 NCO per meiosis although they assessed only 19 and 7, because they did not analyze a whole part of the genome (LOH and chromosomes B and D).

[R] This has been corrected as suggested.

4. Page 10, line 8: refer to Figure 1 at the end of the sentence. Same at the end of this chapter (after “the rest of the genome”).

[R] Figure 1 is now cited at the end of the sentence.

5. Page 14, Discussion: it should be mentioned that in *A. thaliana* and *C. elegans*, the SC polymerization is NOT dependent on ZMM proteins, which may explain why the SC seems to exert a strong effect on CO interference in these species, contrary to budding yeast *S. cerevisiae*.

[R] We have rephrased the sentence and highlighted this in the discussion section.

6. Page 21 legend of Figure 2B: typo: I think p=0.6 should be removed.

[R] Thank you for pointing this error out. We have corrected it.

7. Figure 2B: for clarity, in addition to figure legend, p-values should be added on the graph, for the significant (*S. cerevisiae*) or not significant (*L. kluyveri* and *L. waltii*) inverse relationship between CO density and chromosome size.

[R] Figures have been updated as suggested.
