# Supplementary material for: Lessons from the meiotic recombination landscape of the ZMM deficient budding yeast Lachancea waltii
Source: PLoS Genet. 2023 Jan 6;19(1):e1010592. doi: 10.1371/journal.pgen.1010592 (PMC9851511; doi:10.1371/journal.pgen.1010592)
Supplement: Attachment — Submitted filename: Reviewer_responses_R2_updated.docx [file pgen.1010592.s022.docx]

Dear Michael,

Thank you so much for your feedback on the manuscript. We are happy that the reviewers were in large satisfied with our revision. We addressed the two technical issues as well as the last two points raised by the reviewers. We hope our answers are satisfactory.

Best regards,

Bertrand and Joseph

**Editors comments**

The manuscript was fully evaluated at the editorial level and by independent peer reviewers. The reviewers were in large satisfied with the revision, but both raised points that could be addressed by additional text in a revised manuscript. You should consider this an opportunity that is not obligatory, as addressing these points will add some additional depth to the paper.

1. The formatting of the references is inconsistent, as one would expect for the direct output of a reference manager program. Please make sure that journals are properly abbreviated, that titles are in sentence case, and that all species and gene names are properly italicized.

The references have been updated as per the PLoS Genetics style.

2. Many of the main figures, which currently occupy a full page in landscape orientation, will need to be redone to meet PLOS Genetics figure requirements.

The figures have been redone to fit the PLoS Genetics figure requirements. Additionally, we have split Figures 2 and 3 as described below.

Old figure 2D- New figure 3

Old figures 3A-B- New figures 4A-B

Old figure 3C- New figure 5.

**Reviewer #1**

1. The authors have substantially answered my queries/suggestions. One issue remains.

Hotspot conservation. I appreciate the answer provided in the author response; however, I feel that this general concept needs clearer handling in the text (before lines 249 onwards). Currently this section rolls on directly from the preceding sentences without any introduction. To help the reader, would it be possible to set the scene with a new paragraph here (the concept that hotspots may be conserved with evidence for why this is/was thought to be the case)? Then go on to present the tests/observations that the authors have performed (line 249 onwards).

In general, I find the concept of why there would be crossover hotspot conservation within syntenic blocks between species perplexing. Certainly, in the absence of PRDM9, recombination initiates preferentially in promoter regions where there is low nucleosome occupancy. Yet, I don't understand why this generates any prediction for there to be a cross-species conservation in the *frequency* of recombination arising at orthologous/syntenic regions. Even if the raw sequences were conserved (which I presume they are not), recombination frequency is far more complicated than that—being affected by broad-scale influences such as genomic location, chromosome number and size, proximity to other genomic elements, and by global recombination frequency.

Moreover, unless I am mistaken (and I hope the editor will clarify this with me if I am misrepresenting these prior findings) it has been shown by the editor of this manuscript that even an *identical* piece of DNA has very a different allelic recombination frequency depending on its genomic location (Goldman and Lichten 1996, Table 2).
As such, why then would *diverged* DNA sequences located in different genomic locations/contexts be expected to share similar frequencies? This seems to only make sense if one hypothesizes that the regulators of recombination frequency are both insensitive to the inter-species DNA sequence changes and are also fortuitously constrained to act only within the limits of the syntenic blocks (and thus their influence is conserved). Both seem unlikely. Perhaps this can be explained, discussed and/or refuted? (Perhaps a relevant detail that is missing from the manuscript is how large are the blocks of synteny that have been identified? It seems obvious that the larger the block, the more likely it would be to see similarities in frequency.)

As mentioned in our previous rebuttal, the crossover hotspot conservation issue was addressed in the second paragraph of the introduction. However, it seems that this is not enough according to reviewer 1. So, we have now added a following introductory paragraph (starting at line 227) that we hope will definitely clarify this point and justify our rationale for looking at crossover hotspot conservation between *S. cerevisiae* and other budding yeasts.

“PRDM9 dependent DSB hotspots evolve rapidly compared to PRDM9 independent DSB hotspots that characterize many non-metazoans organisms. PRDM9 independent DSB hotspots mainly depend on DSB formation in functional elements such as gene promoters and were shown to be well conserved in positions and intensities across *Saccharomyces* species. This resulted in the concept of “nonparadoxical evolutionary stability of the recombination initiation landscape in yeast”, mainly explained by the selective pressure on the underlying functional elements determining DSB hotspots (Lam and Keeney 2015). However, this nonparadoxical evolutionary stability is not absolute and its extent is not known. So far, it is restricted to species that show an overall well conserved synteny, while many factors including sequence divergence and synteny rearrangements may affect DSB hotspot strength and location, for instance because of DSB hotspot competition (Wu and Lichten 1995; Robine et al. 2007) and DSB hotspot interference (Garcia et al. 2015). Because DSB hotspots determine crossover hotspots, evaluating the conservation of crossover hotspots between *S. cerevisiae* and *L. waltii* was important to see if the evolutionary stability of the recombination landscape extended beyond the *Saccharomyces* species.”

**Reviewer #2**

The authors satisfactorily addressed all my minor points. However, they did not test the role of Mer3, the sole ZMM protein remaining, for sporulation nor spore viability. I understand that this experimental work could be saved for another manuscript, but I still think it would be important to at least address the point of the possible role (or absence of role) of Mer3 in waltii (for instance in the Discussion).

We have now included in the discussion the following specific paragraph about Mer3, Lines 360-374:

“**Is Mer3 involved in meiotic recombination in *L. waltii*?**

While part of the ZMM dependent crossover pathway, Mer3 likely plays additional roles during meiotic recombination as suggested by the much higher Mer3 foci numbers compared to the observed crossovers numbers in different species (Pyatnitskaya et al. 2019). In addition, in combination with Mlh1-Mlh2, Mer3 limits the extent of DNA repair synthesis during both crossovers and noncrossovers formation in *S. cerevisiae* (Duroc et al. 2017; Vernekar et al. 2021). Because most noncrossovers form independently of the ZMM pathway, this further supports that Mer3 functions extend outside the ZMM pathway. Both Mlh2 and the ZMM proteins Zip2, 3, 4, Spo16, Msh4 and Msh5 being absent in *L. waltii*, this raises the question of any Mer3 meiotic role in this species. Interestingly, we previously reported that Mer3 has been lost four independent times within the *Lachancea* clade, after the loss of Zip2, 3, 4, Spo16, Msh4, 5 and Mlh2 that occurred early, right after the divergence of *L. kluyveri* from the rest of the clade (Vakirlis et al. 2016). Eventually, Mer3 is still present in *L. waltii*, but absent from its closest relative *Lachancea thermotolerans*. One could speculate that these frequent losses of Mer3 along the *Lachancea* phylogeny result from a loss of selective pressure on Mer3. Although this could support an absence of meiotic function of Mer3 in *L. waltii*, this issue needs to be experimentally addressed.”
